# How can rural businesses thrive in the digital economy? A UK perspective^☆^

**DOI:** 10.1016/j.heliyon.2022.e10745

**Published:** 2022-09-25

**Authors:** Pattanapong Tiwasing, Beth Clark, Menelaos Gkartzios

**Affiliations:** a Rural Policy Centre, Scotland's Rural College (SRUC), Edinburgh, EH9 3JG, UK; b Centre for Rural Economy (CRE), Newcastle University, Newcastle upon Tyne, NE1 7RU, UK

**Keywords:** Rural businesses, Digitalisation, Digital economy, United Kingdom

## Abstract

Unlocking the digital potential of the UK's rural areas is important for the future of rural businesses, rural communities and the UK economy as a whole. The use of digital technologies is yielding new opportunities for businesses, including those located rurally, to enhance business growth and economic development, which significantly contributes to UK prosperity. However, businesses, especially small and medium-sized enterprises (SMEs), in rural areas are often digitally disconnected due to inferior digital connectivity and digital exclusion, including lack of internet access and lower levels of digital literacy. Therefore, this paper provides a better understanding of the rural digital economy, highlighting key digital challenges and opportunities for rural businesses in the UK. An extensive review of both academic and non-academic literature is conducted to identify key digital challenges, digital opportunities, and solutions to overcome the digital disadvantage for rural businesses in the UK in the digital age. Our review emphasises the effectiveness of public sector market interventions in developing broadband infrastructure and smarter digital training and skills development to help address digital deprivation in rural areas. A series of policy recommendations is then formulated to support rural business growth in the digital age and contributing to debates regarding smart rural development in rural areas. This paper has potential limitations due to a non-systematic literature review. Therefore, we recommend applying a systematic review as well as empirical and place-based research to explore the emerging themes of this study for future research.

## 1 Why do rural businesses matter?

The purpose of this paper is to review the key barriers and opportunities of rural businesses in the digital era and to improve their digital growth. This matters because rural businesses can play a significant role in achieving the UK's economic objectives, contributing to national economic prosperity and national wellbeing (Phillipson et al., 2019). Rural areas cover the majority of the UK's landmass, although their proportion varies between the different UK nations. For example, rural areas make up 90% of England, whilst 98% of Scotland is considered as rural (House of Lords Library, 2020). In 2018/19, rural areas were a home for approximately 550,000 registered businesses in England (accounting for 23% of all registered businesses) and employing 3.6 million people (13% of all those employed by registered businesses in England) (Department for Environment, Food and Rural Affair (DEFRA), 2021a). Approximately 90% of rural businesses are micro and small businesses. Agriculture is traditionally associated with rural areas; however, it is just one part of an increasingly diversified rural economy. Indeed, the UK's rural economy is broadly similar to the urban economy (OECD, 2011), although policy approaches tackling urban and rural issues can vary considerably because of the association of the rural with farming and agriculture (Gkartzios et al., 2022). For example, a recent DEFRA report (2021a) reveals that in rural areas, agriculture, forestry and fishing sectors accounted for 15.0% of the local units of registered businesses in 2019/20, followed closely by professional, scientific and technical services (14.5%), wholesale, retail and repair of motor vehicles (12.7%), and construction (12.3%). Supporting evidence of the potential of the rural economy, in England alone, rural areas contributed an estimated £261 billion to England's Gross Value Added (GVA), which is approximately a fifth of England's total economy (House of Lords Library, 2020).

Rural businesses also lie at the heart of community services and sustainability and in many cases are seen as drivers of rural development and social enterprise activity (Bosworth and Atterton, 2012; Phillipson et al., 2017; Steiner and Teasdale, 2019). However, they are often unheeded by policy and business support (Phillipson et al., 2019), especially regarding the digital divide (Warren, 2007; Townsend et al., 2016; Tiwasing, 2021). This urban-rural divide mainly occurs from two main issues (Salemink et al., 2017): first, digital connectivity, defined as “*access to fast and reliable internet connection (fixed or mobile) which enables users to benefit from smart and digital services*” (London Assembly Regeneration committee, 2017, p.1); and, secondly, digital inclusion, which refers to “*the activities necessary to ensure that all individuals and communities, including the most disadvantaged, have access to and use of Information and Communication Technologies (ICTs)*” (National Digital Inclusion Alliance, 2021, p.1)”.

Since rural areas can be geographically isolated, in more marginal areas with low population densities (Townsend et al., 2016), this discourages investment in connectivity and digital networks, resulting in the so-called rural digital exclusion, including a lack of digital skills (Warren, 2007; Roberts et al., 2017; Tiwasing, 2021). Despite huge investment in advanced technology in the early 2000s and high-speed broadband highlighted in many research papers and government reports (Williams et al., 2016), access to quality broadband and digital services still remain a major challenge for many rural businesses (Doherty et al., 2016; Philip and Williams, 2019; Rural Service Network, 2021; Currie et al., 2021). In particular, inferior connectivity can restrict rural firms in new technology adoption, participation in innovation activities, business growth and internationalisation, especially in the digital world (Bowen and Morris, 2019). Regarding this point, Wilson et al. (2018) point out that if digital constraints are removed and the digital potential is realised in rural areas, an estimated £15 billion or more could also be generated in additional business turnover per annum and £12 billion of additional GVA per annum to the UK economy. This suggests that rural businesses can and would contribute more, but they need full consideration within economic growth plans and policies in the digital world in order to fulfil their potential.

In this context, it is important to understand the key digital challenges and opportunities in rural areas, in order to improve the digital environment and provide support to rural businesses to thrive in the digital era. Particularly, we draw in this paper on an extensive review of the literature (both grey and academic) in the UK context. We understand that urban-rural binaries vary considerably across the world (and across the UK, see also Gkartzios et al., 2020), but in this paper we draw on the descriptive definitions used by governmental departments distinguishing urban and rural areas (DEFRA, 2016). We highlight key challenges and opportunities as well as policy recommendations to support businesses in rural areas in the UK. Our review also contributes to debates on smart village programme and smart rural development in rural areas.

This paper is structured as follows. Section 2 discusses our research methodology. Section 3 reviews key digital challenges in rural areas and discusses the emergence of digital opportunities for businesses in rural areas. Finally, section 4 discusses how to overcome digital disadvantages in rural areas, with Section 5 detailing the arising policy recommendations.

## 2 Methodology

In this paper, the state-of-the-art review undertaken focuses on the most current information to address the research question on ‘How can rural businesses in the UK thrive in the digital era?’ Thus, to provide an up-to-date information, our review not only focuses on academic research, but also covers grey literature including government reports, policy documents, blogs, news articles and so on. These sources of grey literature are not usually included in a systematic review approach, which may bias perspectives (Grant and Booth, 2009; South and Lorenc, 2020). We acknowledge that our review is *not* systematic, but we also support the view that not all literature reviews need to be systematic (particularly in qualitative research), plus there are also criticisms regarding the use of systematic reviews as a panacea to a review approach (see for example: Petticrew, 2015; Cornish, 2015). We, therefore, apply a traditional literature review to identify key concepts and sources of evidence (both academic and grey literature) to inform practice, policymaking, and research gaps (Daudt et al., 2013), with specific relevance to the UK as our case study country. The UK studies are supplemented with innovative examples from the international literature (e.g., EU, the US, etc.), to provide learnings from other rural communities and governments overcoming digital challenges in rural areas. To answer the research question, our review focuses on three main areas: 1) digital challenges, 2) digital opportunities, and 3) solutions to address the challenges and obtain the opportunities, to inform policy recommendations that support rural business growth in the digital age and to provide future research directions on the rural digital economy.

## 3 Findings and discussion

In this section, we draw on a non-systematic review of both grey and academic literature, which mainly focus on the UK, by identifying (1) digital challenges in rural areas, (2) emerging digital opportunities for rural businesses and (3) solutions to overcome the digital disadvantage in rural areas as presented in the following sections.

### 3.1 The digital challenges in rural areas

The digital economy provides new opportunities for economic and social activities across several domains of life such as economic development, information flow and knowledge transfer, e-commerce, health services, as well as education and training opportunities (Freeman and Park, 2015; Castellacci and Tveito, 2018). It has clear potential to address certain inherent issues with rural geographies, such as geographical remoteness and isolation (Townsend et al., 2016). Rural businesses can gain online access to wider markets and services to improve their competitive position and business performance (Phillipson et al., 2019; Tiwasing, 2021). However, businesses in rural areas in the UK face a number of digital challenges.

For instance, using the information from Ofcom, the House of Commons House of Loads Library (2020) reports that approximately 12% of premises in rural areas do not yet access a basic broadband at 10 Megabit per second (Mbps) internet connection, compared to only 0.4% in urban areas in the UK. More than 20% of premises in rural areas lack superfast broadband connections (download speeds of 30 Mbps), compared to only 2% in urban areas (House of Commons Library, 2020). The European Network for Rural Development (ENRD) (2019) point out that one of the key challenges with rural broadband in the UK is the ‘last mile’**—**the final 5%, which is estimated to have over 1 million premises that are the most critical locations to get connected, especially in the more sparsely populated and peripheral rural areas. In particular, Philip et al. (2017) report that speed and reliability of broadband connection are significantly determined by the ‘last mile’ distance between users and the communications network. If the ‘last mile’ exceeds approximately 1.2 km, superfast broadband that uses cables and copper wires (e.g., Asymmetric Digital Subscriber Lines (ADSL), Fibre broadband, etc) is no longer available and speeds are then limited (Farrington et al., 2015; Philip et al., 2017). Alternatively, gigabit-capable infrastructure, such as 4G and 5G wireless networks, can bring economic and social benefits through enhanced productivity and innovation (Cowie et al., 2020). However, nearly 60% of rural premises live without 4G for all operations compared to only 17% in urban areas (Ofcom, 2018; House of Commons Library, 2020). Therefore, investing in high-speed broadband and advanced networks in rural areas could reduce the rural-urban divide and enhance digital-related activities (Tiwasing, 2021).

Rural businesses often face limited digital service provision due to the high costs of building digital and broadband infrastructure in areas with dispersed geography (Tims, 2020). The low population density of many rural areas is less likely to attract the services of commercial telecoms operators since low population density will reduce the returns that the operators receive from customers taking up services (Tiwasing, 2021). Consequently, this issue adds difficulties for providers in reaching rural premises (and business) cost effectively, resulting in the digital inequalities across the UK and regions (Roberts et al., 2017; Salemink et al., 2017; Philip and Williams, 2019). Lee and Rodríguez-Pose (2013) and Tiwasing et al. (2020) report that London and the South East, which constitute the largest regional UK economy, are often found to have better digital access and networks than other regions. ONS (2019) supports that 93% of population in these regions can access internet, followed by the South West (92.3%) and East of England (92.0%), respectively, while Northern Ireland has the lowest percentage of internet users (86.7%). This digital divide not only affects the everyday activities of people and businesses, but also potentially hinders the development of regional and local economy (Falk and Hagsten, 2021). For example, Atasoy (2013) highlight that access to broadband services is positively associated with employment growth, particularly in rural and remote areas. Also, Tranos et al. (2021) reveal that internet-related technologies have a positively significance on regional productivity in the UK. Therefore, if rural businesses are located in underserved regions, this can create further digital urban-rural divides and then hamper regional and rural economies.

As well as the digital infrastructure and connectivity, rural businesses also struggle to recruit and retain staff with the digital skills needed to help their business thrive and grow (Cosgrave, 2020). In particular, those businesses which do not have in-house digital skills will find it difficult to find external sources of IT support. This lack of digital skills can put many rural enterprises which compete in a market at a disadvantage compared to their urban counterparts (Townsend et al., 2013). Furthermore, rural areas have a greater percentage of older people compared to urban areas, and this age group is often reported to have low levels of digital literacy (Currie et al., 2021; Tiwasing, 2021). Regeneris (2018) reports that more than 50% of the UK population aged between 45 and over 65 years old live in rural areas compared to 39% in urban areas. This poses challenges for rural businesses to recruit young digital-skilled labour (Atterton and Affleck, 2010) and retain the right older workers (Castilla et al., 2018) as well as helping older workers to stay in employment during the digital era (Tiwasing, 2021).

Finally, cyber vulnerabilities, which are the weakness that can be exploited by cybercriminals (Garcia-Perez et al., 2021), are a continued and growing threat for all businesses in the digital era, including rural SMEs (Centre for Rural Enterprise Engagement (CREE), 2021). Miller (2022) reports that rural businesses face similar cyber security challenges to their urban counterparts such as ransomware attacks, database breaches, fraudulent payment requests, and so on. In particular, during the COVID-19 lockdown, the cyber vulnerabilities, including cyber attacks have been significantly increased due to remote work purposes (Jamilov et al., 2021). These spikes can also create an uncertain view of digital business operations in the future with regards to cyber security (Lallie et al., 2021). Using evidence from 400 UK companies, Garcia-Perez et al. (2021) suggest that to effectively manage their risks associated to the digital environment, businesses need to balance the adoption of new technologies and the implementation on the best practices and/or applications related to cyber security in their organisations. Lee (2021) also asserts that implementations of cyber security such as cybertechnology development, testing and monitoring systems, cyber security training, and sufficient budget for necessary cyber response should be put in place and up to date in all organisations. These could help increase awareness among rural businesses (both employees and employers) of cyber security and cyber risks, since digital infrastructure has transformed the way many rural companies do business such as use of email, online accounting tools, online payment systems, online retail activities and so on (Townsend et al., 2016; Shemi and Procter, 2018).

### 3.2 The emerging digital opportunities for rural businesses

Since the UK government has invested considerable sums of public money to address the urban-rural digital divide by improving broadband connectivity and digital infrastructure in rural areas (DEFRA, 2021a, DEFRA, 2021b), the current (and future) connectivity improvements can create digital opportunities for rural businesses in many ways. Firstly, digital technology and connectivity will help rural businesses to address the challenges associated with remoteness (Cowie et al., 2020). Digital technologies also help to reduce costs and improve access to external markets (Phillipson et al., 2019) and enhance economic development in rural areas and local communities (What Works Centre for Local Economic Growth, 2015). Falk and Hagsten (2021) also point out that impact of digital connectivity (e.g., high-speed broadband) on business development is often found to be varied across industries. Such potential benefits are relevant to existing sectors, such as tourism and food and beverage production, but also new sectors wishing to relocate (Townsend et al., 2016).

Secondly, digital tools and services such as e-commerce can play a pivotal role in helping rural businesses to export. According to new findings from Wilson et al. (2018), 80% of rural business owners report that they aim to use e-commerce to trade goods and services internationally. In addition, over 40% of all rural businesses sell their products or services online, either through their own website or a third-party website. In particular, the top two sectors for rural businesses that use e-commerce are the retail (80%) and food and accommodation industries (71%). Furthermore, Townsend et al. (2016) suggest that digital technology and connectivity can offer rural businesses a flexibility of location that helps overcome mobility restrictions. This flexible working pattern (e.g., working from home) can yield important environmental and social benefits alongside the business benefits (Green et al., 2020). In particular, during the COVID-19 pandemic, Ipsen et al. (2021) revealed that working from home can be beneficial for the employees’ wellbeing and performance if organisations put digital and technological support in place for their employees. Townsend et al. (2013) also support that working from home would be beneficial to people who work in rural and remote areas where physical access to the workplace is problematic. Therefore, to support rural homeworking (e.g., videoconferencing, web surfing, email, etc.), this requires decent connectivity networks, particularly upload and download speeds^1^. Ofcom (2016) considers a download speed of at least 10 Mbps and an upload speed of at least 1 Mbps as a decent broadband connection for typical home broadband usage. In fact, such videoconferencing platforms (e.g., Zoom, Teams, livestream video, and so on) require better high-speed connections to continuously operate these online activities for rural households and businesses, which would require upload speeds more than 25 Mbps (Christiansen, 2022). Thus, such high-speed broadband like fibre broadband and/or satellite technology can significantly improve upload speeds (and download speeds) which are essential for videoconferencing as a key part of the new wave of rural homeworking.

In addition, digital technology and broadband connectivity can help to slow down rural-to-urban migration, especially of young people, and bring new jobs and businesses into rural areas (Phillipson et al., 2019). Atterton and Affleck (2010) report that the out-migration of rural youth to urban areas in search of higher education and more lively social environments (see also Stockdale, 2004), is a longstanding issue for rural businesses in seeking to recruit and retain energetic and enthusiastic younger staff. Also, these economically active rural residents can help support local shops and community activities, helping to safeguard rural services. If inferior digital connectivity and infrastructure in rural areas are fixed, rural businesses could be more profitable than those in urban areas because of fewer fixed costs related to business premises and lower costs for labour, particularly compared to large urban centres (Phillipson et al., 2019).

Moreover, digital technology can facilitate online co-working space and business networking activities, giving rural businesses a chance to collaborate and exchange knowledge (Merrell et al., 2021; Merrell et al., 2022). Quinton and Wilson (2016) also emphasise that participating in online business networks through social media networks can quickly build trustworthiness within the networks, contributing to business engagement and collaborative problem solving. Using the UK Government's Longitudinal Small Business Survey, Tiwasing (2021) also reveals that being members of online business networks (e.g., LinkedIn) can help rural businesses enhance their business performance and sales growth in England and Wales. This stresses the importance of online business networking activities and digital social capital on business performance development, and rural businesses should be encouraged to make use of these online activities to gain more business benefits, such as external training, external finance, funding support, and so on (Mole et al., 2017).

Finally, in recent years, digital hubs are recommended as a digital operating model to enhance the local digital environment and build digital collaborative communities that foster both social connectivity and economic change in rural areas (Price et al., 2018; Rundel et al., 2020; Merrell et al., 2021, Merrell et al., 2022). They are often used as co-working spaces to attract and retain digital entrepreneurs and young talent (Price et al., 2021). In the UK, digital and technological hubs have recently been established to provide businesses with access to technical capabilities, equipment and other services to create innovation capability and economic growth (Innovate UK, 2020). In particular, these digital hubs are often a collaboration by businesses, universities and local government agencies as part of regional and local development funding (Price et al., 2021; Rundel and Salemink, 2022). For example, the Lincolnshire Technology Hubs in the UK is located in the University of Lincoln to provide businesses with access to digitally-enabled technology, which can be used by the local manufacturing sector, and digital skills support and advice (Ashmore et al., 2019). Another successful example is the Centre for Digital Innovation (C4DI) in Hull (UK), which is as an incubator and co-working space to help boost digital skills to create more jobs and investment opportunities in the area (York & North Yorkshire Local Enterprise Partnership, 2020). However, these digital hubs are often located in larger cities and urban areas, which could be difficult for businesses in rural areas to access, especially with current cost of travel (Merrell et al., 2021, Merrell et al., 2022). Therefore, if these digital hubs are established in rural areas, rural firms can use them as the catalyst of a whole range of digital-related initiatives and activities to build social capital and networks in rural communities and enhance business performance and growth (Rundel et al., 2020).

### 3.3 Overcoming the digital disadvantage in rural areas

To address the digital deprivation in rural locations, strategies should create the conditions for better digital access to ensure digital benefits for rural businesses and communities. Learning from EU policy development, the ENRD (2018) introduced the concept of “Smart Villages” to promote the role of innovation (both technological and social innovation) for rural development and the resilience of rural places within the networks. This was also supported by the EU Action for Smart Villages initiative, embracing more sophisticated and multi-sectoral policies than just the development of rural digital services. For example, it is not only targeting digital investment, but also aiming to build digital skills and, wider, digital coordinated governance in rural areas (Gkartzios et al., 2022). In the UK, the Smart Villages model has first been implemented in Cornwall as a pilot project to develop digital strategies for tackling issues of rural isolation, ageing population and low business productivity through collaborating between local and regional stakeholders from both the public and private sectors (ENRD, 2019).

**Figure 1:**
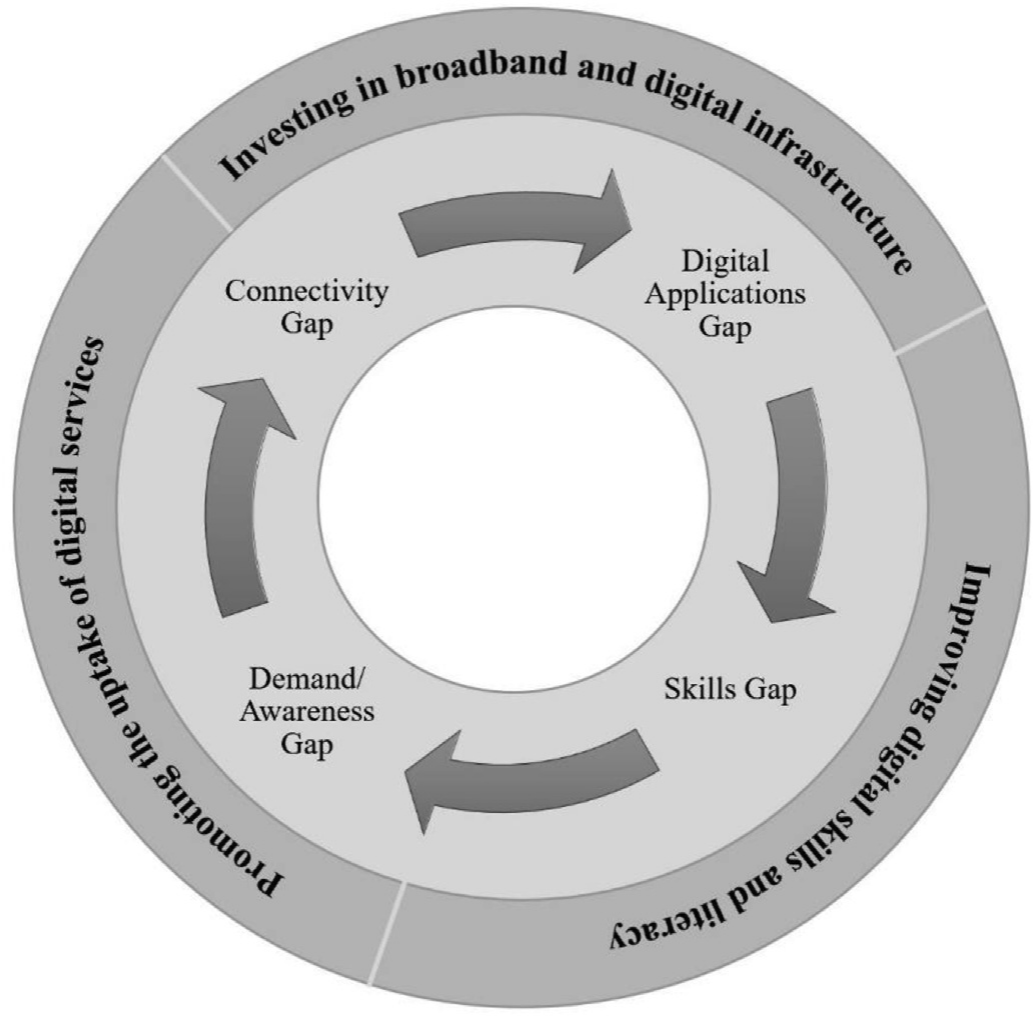
Links between connectivity, digital applications, skills and demand gaps. *Source*: Authors, adapted from ENRD (2018).

Although this model has now expanded to some parts of the UK (e.g., Smart Village Scotland), a number of UK initiatives are still focused on harnessing the power of technology for rural places (Cowie et al., 2020). Therefore, to ensure digital strategies benefit rural businesses and support the transition to “smart rural” futures in the UK, much scholarship (e.g., Warren, 2007; Townsend et al., 2013; ENRD, 2018; Phillipson et al., 2019; Cowie et al., 2020) has pointed out that strategies should consider the three components of the digital divide in parallel with the specific needs of each rural area, and the existing landscape of policy support, which is: 1) Investing in broadband and digital infrastructure; 2) Improving digital skills and literacy; and, 3) Promoting the uptake of digital services. Figure 1 reveals how these three components reinforce each other.

In Figure 1, the inside circle presents the digital problems in rural areas, while the outside circle is the suggested solutions to address these problems. To narrow down the rural-urban digital connectivity gap, an investment in high-speed broadband and quality digital infrastructure (e.g., superfast broadband, fibre broadband, satellite technology) in rural areas is key (Williams et al., 2016; Philip et al., 2017). In particular, satellite broadband is a better option for acquiring internet access in rural places where 4G and a mobile broadband are difficult to find (Beckett, 2021), particularly very remote areas like Scotland's Highlands and Islands (Ofcom, 2020). Satellite technology can also be particularly useful in connecting very remote communities where laying cables (e.g., superfast and fibre broadband) is not possible (Rogers, 2021). However, Beckett (2021) suggests that if those rural places are covered by 4G (or 5G), it might be better off for rural households and businesses to look for a mobile broadband deal, since they are cheaper and faster than a standard satellite connection due to satellite-supported technologies and provisions including the need to invest in a satellite dish and costs of its' installation. Townsend et al. (2016) also support that investing in the right gadget and technology for digital connectivity can help reduce the digital divide between urban and rural locations sustainably, providing rural businesses with better opportunities to access digital-related activities and applications (Tiwasing et al., 2020; Tiwasing, 2021). However, successful digital transformation for rural businesses in the UK requires much more support than the improvement of digital infrastructure and broadband services (Cowie et al., 2019).

Businesses in rural areas are particularly vulnerable to the digital exclusion, because of the limitations on skills and capabilities relating to digital technology (Warren, 2007; Townsend et al., 2016). If they do not have human capital with essential digital skills and do not know how to use digital technologies effectively, this would lead to a low level of digital awareness, which in turn damages the business case for further investments (Williams et al., 2016; Phillipson et al., 2019). Therefore, to help rural businesses to improve these disadvantages, digital support services and appropriate training should be provided to businesses in rural areas, especially those who rely on digital services and online operations (Tiwasing, 2021). Moreover, rural businesses are often unaware of the benefits of digital services and applications (e.g., e-commerce, online booking system, social media networks, etc.) that can dramatically improve their businesses (Williams et al., 2016; Wilson et al., 2018). In particular, online sales, especially e-commerce have now played a vital role in the UK economy with 33.9% of total retail sales during the COVID-19 pandemic (ONS, 2021). Thus, Government and business support agencies should focus on promoting the uptake of digital services and applications by delivering easily accessible advice services and showcasing how to use suitable digital applications for difference businesses in order to raise digital awareness among rural businesses in the digital age (ENRD, 2018).

## 4 Conclusion and recommendations for rural business growth in the digital age

This paper builds on an up-to-date literature review to respond to the question on “how rural business in the UK can thrive in the digital age?”. Our review focuses on three primary aspects in order to answer this question, which are digital challenges, digital opportunities, and solutions to address those challenges and realise the opportunities of rural areas. This review highlights the lack of decent and suitable broadband connections, digital service provision limitations and ‘last mile’ connectivity, low level of digital skills and knowledge, difficulties in retaining and recruiting young digital-skilled labour, and cyber vulnerabilities as the main digital challenges in rural UK. This review also reports the emerging digital opportunities to support rural businesses in the UK, via e-commerce and online services, flexible working patterns, online co-working spaces, online networking activities and collaborative communities, which also help overcome prolonged rural problems such as tackling rural-to-urban migration across young people, and so on. To obtain the full potential of these digital opportunities, our review provides a framework developed from the “Smart Village” concept to help overcome digital disadvantages. It emphasises the reinforcement between digital infrastructure investment, digital skills improvement, and promoting the uptake of digital services.

This review is helpful for policy recommendations to support rural business growth in the digital age and for future research directions on rural digital economies. In particular, the rapid growth of the digital economy and particularly the COVID-19 pandemic have accelerated the spread of digital technology use, leading to a pressing need for businesses and governments to adapt. Many rural businesses have been digitally disadvantaged due to the digital divide and associated exclusion. They may struggle to find employees with the right skills and face challenging business conditions. In addition, labour in rural areas lacks the skills or flexibility in labour markets to adapt. This suggests that rural businesses need to embrace technology and upgrade training programmes to equip their workers with the right sets of skills.

So far, most of the digital policies and debates related to rural areas in the UK have concentrated on the issue of access to infrastructure, specifically connectivity to high-speed broadband and mobile networks (Williams et al., 2016), since many rural premises are still unable to access decent network speeds or reliability necessary for practical everyday use (Warren, 2007; Townsend et al., 2016; DEFRA, 2021b; Tiwasing, 2021). Additionally, the UK's Government funded initiatives (i.e., UK Gigabit Programme, Superfast Broadband Programme, Universal Service Obligation Programme, Gigabit Broadband Voucher Scheme, etc.) have attempted to reduce the digital divide in the country. In particular, these programmes primarily aim to expand broadband access in under-served rural areas, which are similar to the government digital support programmes in other developed countries such as USDA's Broadband Loan Programme in the US (see Kandilov and Renkow, 2010; Dinterman and Renkow, 2017) and State Aid for broadband in the EU (see Bourreau et al., 2020). Yet, hundreds of thousands of premises in rural and “hard-to reach” areas are still in need of decent (10 Mbps), superfast (30 Mbps), and fibre (up to around 35 Mbps or 60 Mbps) broadband speeds (DEFRA, 2021a, b). Therefore, the initiatives to boost digital infrastructure and connectivity are still likely to be a significant policy concern. However, successful technological improvement requires a critical mass of financial, human and social capital (Cowie et al., 2019; Rural Service Network, 2021). Currently, the UK Government's Digital Strategy, led by the Department for Digital, Culture, Media & Sport (DCMS), aims to help every business in the UK become a “digital business” by looking beyond the improvement of digital tools and connectivity such as access to the digital skills support and training, building strong technology sectors and digital ecosystems and so on (DCMS,2022). This emphasises the need to ensure that rural businesses are not left behind. Therefore, during rapid digital transformation, UK policy should not only consider an urgent investment in digital connectivity and networks for rural businesses who are under-serviced by digital services, but also the enhancement of digital skills and literacy and promoting the uptake of new digital innovations (Rural Service Network, 2021). More importantly, the government support programme should also consider the suitable types of broadband connections for specific geographical locations (such as satellite technology for some rural and remote areas, where cable networks and full-fibre technologies are not available) in order to provide adequate broadband, and keep rural households and businesses connected with decent network services as well as reducing the high cost of provision in very remote areas.

Drawing on our review, to support UK business in rural areas thrive in the digital world, we can sum up the following recommendations for the private and public sectors to support rural businesses. First of all, to solve territorial digital inequalities and provide low-cost digital and broadband services for rural enterprises, digital investments need to ensure the effectiveness of the public sector's market interventions in broadband infrastructure developments (Williams et al., 2016), especially those who rely on the use of digital services, such as the tourism and food/drink sectors (Townsend et al., 2016). Secondly, our review points to the catalytic role of smarter digital training and skills development through local/regional government agencies. This can help rural businesses to improve the level of digital skills within their businesses, for both employees and business owners, to meet their digital needs (Tiwasing, 2021). In addition, simpler signposting to digital support and IT information, such as local guidance, should be created for rural businesses seeking appropriate digital and IT support (Salemink et al., 2017). Similarly, it is important to create more digital enterprise hubs for better access to digital support in rural areas (Rundel et al., 2020; Price et al., 2021). Rural businesses can use or visit the hubs for better connectivity, start-up workspace, hot-desk space and digital training (Rundel et al., 2020; Merrell et al., 2021, Merrell et al., 2022).

Next, faster business adoption of digital connectivity is needed by raising superfast broadband uptake by rural businesses, by reinforcing efforts to promote the business benefits, and encouraging those already using superfast broadband to champion it to their peers in order to provide real world examples of the benefits (Cowie et al., 2020). In that regard, identifying “Digital Champions” in local communities is paramount (ENRD, 2018). Moreover, e-commerce and online retail activities should be promoted to create internationalisation and more marketing opportunities for rural businesses (Tiwasing, 2021), including the option of using third party e-commerce websites. Finally, from a strategic perspective, reconsidering existing policies and strategies with a stronger rural focus is essential to support digital growth and investment for the future of rural businesses, including post-Brexit and the COVID pandemic (Phillipson et al., 2020; Currie et al., 2021). Such policies can include a digital-related fund, building digital infrastructure support, digital skill development/training, and online business advice and IT support to help rural businesses become a digital business. This support should also consider the improvement of the cyber security environment to make the UK the safe place to live in and work online, since rural businesses are using more digital technologies (e.g., online accounting tools, email, digital payment solutions, etc.) and agricultural innovations (e.g., driverless tractors, automated milking machines, and harvesting robots, etc.). This will inevitably increase the cyber security threats. To help address these issues, the UK government's National Cyber Security Centre (NCSC, 2021a, NCSC, 2021b) has now offered a wealth of cyber security guidance and other helpful online resources (e.g., free training course, cyber advisor, cyber essentials certification, etc.) for UK businesses, including rural SMEs and those in agricultural and farming sectors (NCSC, 2021a, NCSC, 2021b; The Northern Irish Department of Agriculture, Environment and Rural Affairs (DAERA), 2022). Therefore, rural businesses and residents, especially older people and those who are vulnerable to cyber threats, should be encouraged to use these cyber security services and advice to help them achieve a minimum standard of security and guard their businesses and everyday online activities against cyber attacks.

Our review also provides implications for business owners and practitioners. Firstly, given the importance of e-commerce and internationalisation, rural businesses should incorporate e-commerce with their retail activities and business strategies in order to expand to new markets nationally and internationally, especially in the digital era and post COVID. Secondly, rural businesses should participate in online business networks and/or visit digital hubs to gain external information and exchange knowledge. Participating in online and offline networking activities would also help rural businesses to build business relationships and expand their business connections and networks. In addition, rural businesses should invest in On-The-Job-Training, particularly digital skills and literacy for different levels of employees to improve labour digital competence in their workplaces. Finally, when high-speed broadband services are available, rural businesses in those areas should be ready to invest in and uptake advanced technologies to ensure that they can fully seize the benefits of the digital age. This could also help them to attract and retain young talent and digital entrepreneurs in rural areas.

## 5 Future research

This paper suggests avenues for future research. Firstly, since our review only focus on the UK context, future research would be beneficial by comparing the current situation of rural digital economy in the UK with other countries. Next, this paper does not apply a systematic review since we aim to provide up-to-date evidence and specific emerging themes on how to help rural business flourish in the digital era such as smart rural, online co-working space, and cyber security in rural areas and so on. Thus, it would be interesting for future research to apply a systematic review approach (see Denyer and Tranfield, 2009; Fisch and Block, 2018; Xiao and Watson, 2019) to explore these emerging themes. Finally, future research should apply quantitative and/or qualitative analysis, including a synthesis of technical and socio-economic approaches, to provide evidence-based recommendations related to these emerging themes to help support rural businesses in the digital economy.

## Declarations

### Author contribution statement

Pattanapong Tiwasing, Beth Clark, Menelaos Gkartzios: conceived and designed the experiments; analyzed and interpreted the data; contributed reagents, materials, analysis tools or data; wrote the paper.

### Funding statement

This work was supported by the Novel Insights on Scotland’s Rural and Island Economies (NISRIE) project under the 2022-2027 Strategic Research Programme of the Scottish Government’s Rural and Environment Science and Analytical Services (RESAS) Division [RESAS 22-27: SRUC-E1-1].

### Data availability statement

No data was used for the research described in the article.

### Declaration of interest’s statement

The authors declare no conflict of interest.

### Additional information

No additional information is available for this paper.

## Footnotes

1 Following Ofcom (2016, p. 7), download speed refers to “the speed (megabits per second (Mbps)) at which a connection can download data from the internet to a computer, laptop, tablet or other device”. While upload speed refers to “the speed (Mbps) at which a connection can transfer data from a device to the internet.”

## References

[bib1] Ashmore F., Price L., Deville J. (2019). https://eprints.lincoln.ac.uk/id/eprint/41175/1/CORA_Digital_Hub_Guide_14.01.2020_Executive_Summary%20%281%29.pdf.

[bib2] Atasoy H. (2013). The effects of broadband internet expansion on labor market outcomes. ILR Rev..

[bib91] Atterton J., Affleck A. (2010). Rural businesses in the North East of England: final survey results (2009). https://www.ncl.ac.uk/media/wwwnclacuk/centreforruraleconomy/files/ne-survey-results.pdf.

[bib3] Beckett M. (2021). Satellite internet explained: is satellite broadband any good?. https://www.uswitch.com/broadband/guides/satellite-broadband/.

[bib4] Bosworth G., Atterton J. (2012). Entrepreneurial in-migration and Neoendogenous rural development. Rural Sociol..

[bib5] Bourreau M., Feasey R., Nicolle A. (2020). Assessing fifteen years of State Aid for broadband in the European Union: a quantitative analysis. Telecommun. Pol..

[bib6] Bowen R., Morris W. (2019). The digital divide: implications for agribusiness and entrepreneurship. Lessons from Wales. J. Rural Stud..

[bib7] Castellacci F., Tveito V. (2018). Internet use and well-being: a survey and a theoretical framework. Res. Pol..

[bib8] Castilla D., Botella C., Miralles I., Bretón-López J., Dragomir-Davis A.M., Zaragoza I., Garcia-Palacios A. (2018). Teaching digital literacy skills to the elderly using a social network with linear navigation: a case study in a rural area. Int. J. Hum. Comput. Stud..

[bib9] CREE (2021). Cree’s Blog Posts.

[bib10] Christiansen P. (2022). What is a good download and upload speed?. https://www.highspeedinternet.com/resources/what-is-a-good-download-upload-speed.

[bib11] Cornish F. (2015). Evidence synthesis in international development: a critique of systematic reviews and a pragmatist alternative. Anthropol. Med..

[bib12] Cosgrave C. (2020). The whole-of-person retention improvement framework: a guide for addressing health workforce challenges in the rural context. Int. J. Environ. Res. Publ. Health.

[bib13] Cowie P., Tiwasing P., Phillipson J., Gorton M., Scott M., Gallent N., Gkartzios M. (2019). The Routledge Companion to Rural Planning.

[bib14] Cowie P., Townsend L., Salemink K. (2020). Smart rural futures: will rural areas be left behind in the 4th industrial revolution?. J. Rural Stud..

[bib15] Currie M., McMorran R., Hopkins J., McKee A.J., Glass J., Wilson R., Meador J.E., Noble C., Craigie M.C., Piras S., Bruce F. (2021). “Understanding the Response to Covid-19-Exploring Options for a Resilient Social and Economic Recovery in Scotland’s Rural and Island Communities”, Working Paper, SAFARI, the James Hutton Institute and Scotland’s Rural College. https://sefari.scot/document/rural-and-island-communities-response-to-covid-19.

[bib16] DAERA (2022). New Cybersecurity Course for Farm Families”. https://www.daera-ni.gov.uk/news/new-cybersecurity-course-farm-families.

[bib17] Daudt H.M., van Mossel C., Scott S.J. (2013). Enhancing the scoping study methodology: a large, inter-professional team's experience with Arksey and O'Malley's framework. BMC Med. Res. Methodol..

[bib18] DEFRA (2016). Rural urban Classification. https://www.gov.uk/government/collections/rural-urban-classification.

[bib23] DCMS (2022). Building Digital UK”. https://www.gov.uk/guidance/building-digital-uk.

[bib19] DEFRA (2021). “Statistics Digest of Rural England”. https://assets.publishing.service.gov.uk/government/uploads/system/uploads/attachment_data/file/984879/Businesses_March_2021_final_with_cover_page.pdf.

[bib20] DEFRA (2021). “Statistical digest of rural England: broadband”. https://assets.publishing.service.gov.uk/government/uploads/system/uploads/attachment_data/file/996572/Broadband_June_2021_final_with_cover_page.pdf%20(6.

[bib21] Denyer D., Tranfield D., Buchanan D.A., Bryman A. (2009). The Sage Handbook of Organizational Research Methods.

[bib22] Dinterman R., Renkow M. (2017). Evaluation of USDA’s broadband loan program: impacts on broadband provision. Telecommun. Pol..

[bib24] Doherty E., Ramsey E., Harrigan P., Ibbotson P. (2016). Impact of broadband internet technologies on business performance of Irish SMEs. Strat. Change.

[bib25] ENRD (2018). “Smart Villages – How to Ensure that Digital Strategies Benefit Rural Communities: Orientations for Policymakers and Implementers”. https://enrd.ec.europa.eu/sites/default/files/enrd_publications/smart-villages_orientations_digital-strategies.pdf.

[bib26] ENRD (2019). Cornwall-UK: Steps towards a digital rural region. https://enrd.ec.europa.eu/sites/default/files/enrd_publications/digital-strategies_case-study_uk_0.pdf.

[bib27] Falk M., Hagsten E. (2021). Impact of high-speed broadband access on local establishment dynamics. Telecommun. Pol..

[bib28] Farrington J., Philip L., Cottrill C., Abbott P., Blank G., Dutton W.H. (2015). Two-speed Britain: rural internet use. https://ssrn.com/abstract=2645771.

[bib29] Fisch C., Block J. (2018). Six tips for your (systematic) literature review in business and management research. Manag. Rev. Quart..

[bib30] Freeman J., Park S. (2015). Rural realities: digital communication challenges for rural Australian local governments. Transforming Gov. People, Process Policy.

[bib31] Garcia-Perez A., Sallos M.P., Tiwasing P. (2021). Dimensions of cybersecurity performance and crisis response in critical infrastructure organisations: an intellectual capital perspective. J. Intellect. Cap..

[bib32] Gkartzios M., Toishi N., Woods M. (2020). The language of rural: reflections towards an inclusive rural social science. J. Rural Stud..

[bib33] Gkartzios M., Gallent N., Scott M. (2022).

[bib34] Grant M.J., Booth A. (2009). A typology of reviews: an analysis of 14 review types and associated methodologies. Health Info. Librar. J..

[bib35] Green N., Tappin D., Bentley T. (2020). Working from home before, during and after the Covid-19 pandemic: implications for workers and organisations. New Zealand J. Employment Relations.

[bib36] House of Commons Library (2020). Building the UK’s digital future. https://commonslibrary.parliament.uk/building-the-uk-digital-future/.

[bib37] House of Loads Library (2020). Fact file: rural economy. https://lordslibrary.parliament.uk/fact-file-rural-economy/.

[bib38] House of Lords Library (2020). “Rural Economy and UK Agriculture: Issues for the New Parliament”. https://lordslibrary.parliament.uk/research-briefings/lln-2020-0029/.

[bib39] Innovate UK. (2020). “Creating the Future through Innovation Network: Recovery and Resilience”. https://catapult.org.uk/wp-content/uploads/2020/12/Catapult-Network-Impact-Brochure-2020-FINAL.pdf.

[bib40] Ipsen C., van Veldhoven M., Kirchner K., Hansen J.P. (2021). Six key advantages and disadvantages of working from home in Europe during COVID-19. Int. J. Environ. Res. Pub. Health.

[bib41] Jamilov R., Rey H., Tahoun A. (2021). https://www.nber.org/papers/w28906.

[bib42] Kandilov I.T., Renkow M. (2010). Infrastructure investment and rural economic development: an evaluation of USDA's broadband loan program. Growth and Change.

[bib43] Lallie H.S., Shepherd L.A., Nurse J.R.C., Erola A., Epiphaniou G.M., Maple C., Bellekens X. (2021). Cyber security in the age of COVID-19: a timeline and analysis of cyber-crime and cyber-attacks during the pandemic. Comp. Security.

[bib44] Lee I. (2021). Cybersecurity: risk management framework and investment cost analysis. Business Horizons.

[bib45] Lee N., Rodríguez-Pose A. (2013). Original innovation, learnt innovation and cities: evidence from UK SMEs. Urban Stud..

[bib46] London Assembly Regeneration committee (2017). Digital Connectivity in London”. https://www.london.gov.uk/sites/default/files/digital_connectivity_report_final.pdf.

[bib47] Merrell I., Füzi A., Russell E., Bosworth G. (2021). How rural coworking hubs can facilitate well-being through the satisfaction of key psychological needs. Local Econ..

[bib48] Merrell I., Phillipson J., Gorton M., Cowie P. (2022). Enterprise hubs as a mechanism for local economic development in rural areas. J. Rural Stud..

[bib49] Miller A. (2022). “How Rural Businesses Can Overcome Their Cyber Security Handicaps”. https://www.itpro.co.uk/security/368269/how-rural-businesses-can-overcome-cyber-security-handicaps.

[bib93] Mole K., North D., Baldock R. (2017). Which SMEs seek external support? Business characteristics, management behaviour and external influences in a contingency approach. Environment and Planning C: Politics and Space.

[bib50] National Digital Inclusion Alliance (2021). “Definitions. National Digital Inclusion Alliance”. https://www.digitalinclusion.org/definitions.

[bib51] NCSC (2021). Cyber security for farmers. https://www.ncsc.gov.uk/guidance/cyber-security-for-farmers.

[bib52] NCSC (2021). Cyber advisor. https://www.ncsc.gov.uk/information/cyber-advisor.

[bib53] OECD (2011).

[bib54] Ofcom (2016). UK Home broadband performance: A consumer summary of fixed-line broadband performance provided to residential consumers”. https://www.ofcom.org.uk/__data/assets/pdf_file/0030/78267/fixed-bb-speeds-nov15-consumer-summary.pdf.

[bib55] Ofcom (2018). https://www.ofcom.org.uk/__data/assets/pdf_file/0019/130681/Economic-Geography-2018.pdf.

[bib56] Ofcom (2020). “Connected Nations 2019: Scotland report”. https://www.ofcom.org.uk/__data/assets/pdf_file/0028/186409/connected-nations-2019-scotland-report.pdf.

[bib57] ONS (2019). “Internet users. https://www.ons.gov.uk/businessindustryandtrade/itandinternetindustry/bulletins/internetusers/2019.

[bib58] ONS (2021). “Impact of the Coronavirus (COVID-19) Pandemic on Retail Sales in 2020”. https://www.ons.gov.uk/economy/grossdomesticproductgdp/articles/impactofthecoronaviruscovid19pandemiconretailsalesin2020/2021-01-28.

[bib59] Petticrew M. (2015). Time to rethink the systematic review catechism? Moving from ‘what works’ to ‘what happens. System. Rev..

[bib60] Philip L., Williams F. (2019). Remote rural home based businesses and digital inequalities: understanding needs and expectations in a digitally underserved community. J. Rural Stud..

[bib61] Philip L., Cottrill C., Farrington J., Williams F., Ashmore F. (2017). The digital divide: patterns, policy and scenarios for connecting the ‘final few’in rural communities across Great Britain. J. Rural Stud..

[bib62] Phillipson J., Gorton M., Maioli S., Newbery R., Tiwasing P., Turner R. (2017). “Small Rural Firms in English Regions: Analysis and Key Findings from the UK Longitudinal Small Business Survey”, Working Paper, Newcastle University’s Centre for Rural Economy and Business School. https://eprints.ncl.ac.uk/file_store/production/239891/53BBF6B6-67BD-4416-A5DB-361E2CF7C2D5.pdf.

[bib63] Phillipson J., Gorton M., Turner R., Shucksmith M., Aitken-McDermott K., Areal F., Cowie P., Hubbard C., Maioli S., McAreavey R., Souza-Monteiro D. (2020). The COVID-19 pandemic and its implications for rural economies. Sustainability.

[bib64] Phillipson J., Tiwasing P., Gorton M., Maioli S., Newbery R., Turner R. (2019). Shining a spotlight on small rural businesses: how does their performance compare with urban?. J. Rural Stud..

[bib65] Price L., Deville J., Ashmore F. (2021). A guide to developing a rural digital hub. Local Econ..

[bib66] Price L., Shutt J., Sellick J. (2018). Supporting rural small and medium-sized Enterprises to take up broadband-enabled technology: what works?. Local Econ..

[bib67] Quinton S., Wilson D. (2016). Tensions and ties in social media networks: towards a model of understanding business relationship development and business performance enhancement through the use of LinkedIn. Industrial Marketing Management.

[bib68] Regeneris (2018). “The Impact of High-Speed Broadband for Communities”. https://www.bt.com/bt-plc/assets/documents/about-bt/bt-uk-and-worldwide/bt-in-the-uk-and-ireland/research-and-reports/the-impact-of-high-speed-broadband-for-communities.pdf.

[bib69] Roberts E., Beel D., Philip L., Townsend L. (2017). Rural resilience in a digital society: Editorial. J. Rural Stud..

[bib70] Rogers A. (2021). Is fiber internet or satellite internet the future?. https://rcrwireless.com/20211210/opinion/readerforum/is-fiber-internet-or-satellite-internet-the-future-reader-forum.

[bib71] Rundel C., Salemink K. (2022). “Hubs, hopes and high stakes for a relatively disadvantaged low tech place”. Local Econ..

[bib72] Rundel C.T., Salemink K., Strijker D. (2020). Exploring rural digital hubs and their possible contribution to communities in Europe. J. Rural Comm. Develop..

[bib73] Rural Service Network (2021). Are rural areas falling through the net?. https://www.rsnonline.org.uk/are-rural-areas-falling-through-the-net.

[bib74] Salemink K., Strijker D., Bosworth G. (2017). Rural development in the digital age: a systematic literature review on unequal ICT availability, adoption, and use in rural areas. J. Rural Stud..

[bib90] Shemi A.P, Procter C. (2018). E-commerce and entrepreneurship in SMEs: case of myBot. Journal of Small Business and Enterprise Development.

[bib75] South E., Lorenc T. (2020). Use and value of systematic reviews in English local authority public health: a qualitative study. BMC Pub. Heal..

[bib76] Steiner A., Teasdale S. (2019). Unlocking the potential of rural social enterprise. J. Rural Stud..

[bib77] Stockdale A. (2004). Rural out-migration: community consequences and individual migrant experiences. Sociologia Ruralis.

[bib78] Tims A. (2020). “BT Broadband Bills Could Reach £100,000 for Rural Users”, the Guardian, 14 September. https://www.theguardian.com/money/2020/sep/14/bt-broadband-bills-could-reach-100000-for-rural-users.

[bib79] Tiwasing P. (2021). Social media business networks and SME performance: a rural–urban comparative analysis. Growth and Change.

[bib80] Tiwasing P., Kim Y.R., Akinremi T. (2020). Spatial disparities in SME productivity: evidence from the service sector in England. Reg. Stud. Reg. Sci..

[bib81] Townsend L., Sathiaseelan A., Fairhurst G., Wallace C. (2013). Enhanced broadband access as a solution to the social and economic problems of the rural digital divide. Local Econ..

[bib82] Townsend L., Wallace C., Smart A., Norman T. (2016). Building virtual bridges: how rural micro-enterprises develop social capital in online and face-to-face settings. Sociologia Ruralis.

[bib83] Tranos E., Kitsos T., Ortega-Argilés R. (2021). Digital economy in the UK: regional productivity effects of early adoption. Reg. Stud..

[bib84] Warren M. (2007). The digital vicious cycle: Links between social disadvantage and digital exclusion in rural areas. Telecomm. Pol..

[bib85] What works centre for local economic growth (2015). “Broadband”. http://www.whatworksgrowth.org/policy-reviews/broadband/.

[bib86] Williams F., Philip L., Farrington J., Fairhurst G. (2016). Digital by Default’and the ‘hard to reach’: exploring solutions to digital exclusion in remote rural areas. Local Economy.

[bib87] Wilson B., Atterton J., Hart J., Spencer M., Thomson S. (2018). Unlocking the Digital Potential of Rural Areas across the UK”, Report, Rural England and Scotland’s Rural College. https://ruralengland.org/wp-content/uploads/2018/03/Unlocking-digital-potential-website-version-final.pdf.

[bib88] Xiao Y., Watson M. (2019). Guidance on conducting a systematic literature review. J. Plann. Educ. Res..

[bib89] York & North Yorkshire Local Enterprise Partnership (2020). “Treadmills Delivers Economic Boost as First Phase Opens to the Public”. https://www.ynylep.com/news/news-articles/details/treadmills-delivers-economic-boost-as-first-phase-opens-to-the-public.

